# Cytokine generation, promoter activation, and oxidant-independent NF-κB activation in a transfectable human neutrophilic cellular model

**DOI:** 10.1186/1471-2172-9-14

**Published:** 2008-04-11

**Authors:** Thornin Ear, Patrick P McDonald

**Affiliations:** 1 Pulmonary Division, Faculty of Medicine, Université de Sherbrooke and Centre de recherche du CHUS, Sherbrooke, Qc, Canada

## Abstract

**Background:**

Human neutrophils are key players of innate immunity, and influence inflammatory and immune reactions through the production of numerous cytokines and chemokines. Despite major advances in our understanding of this important functional response of neutrophils, the short lifespan of these cells and their resistance to transfection have always been an obstacle to the detailed dissection of signaling pathways and effector responses that is often possible in other cell types.

**Results:**

Here, we report that granulocytic differentiation of human PLB-985 cells with DMSO yields cells that are neutrophil-like with respect to surface markers, acquisition of responsiveness to physiological neutrophil stimuli (fMLP, LPS), cytokine expression and production profile, and transcription factor activation profile (NF-κB, C/EBP, AP-1, STAT). We also show that granulocytic PLB-985 cells can be reliably tranfected by nucleofection in a rapid and efficient manner. Indeed, we overexpressed several proteins and luciferase constructs into these cells. In particular, overexpression of a dominant negative IκB-α confirmed the central role of NF-κB in the production of cytokines by granulocytes. Moreover, the use of PLB-985 granulocytes in which the NADPH oxidase is inactive due to the targeted disruption of a key component (gp91phox) revealed that NF-κB activation and κB-dependent responses are independent of endogenous reactive oxygen intermediates in these cells. Antioxidant studies performed in primary human neutrophils support this conclusion.

**Conclusion:**

Our results unveil a new facet of the NF-κB system of human granulocytes, and pave the way for deciphering signal transduction pathways and promoter activation in these cells.

## Introduction

Human polymorphonuclear neutrophils are terminally differentiated cells that represent about 60% of all circulating leukocytes. Beside their notorious role as professional phagocytes, neutrophils can also express a wide array of cytokines and chemokines in response to physiological stimuli [1]. In this regard, a mounting body of evidence shows that neutrophil-derived cytokines and chemokines play an important role in several inflammatory and immune reactions *in vivo *[2-8]. Although much progress has been made in understanding the various facets of neutrophil biology, studies of the signal transduction mechanisms leading to functional activation of neutrophils have remained hampered by the fact that these cells are very refractory to transfection. This owes much to the fact that their lifespan merely averages 16–24 h in the bloodstream [9]. Thus, the characterization of a cell line that can be differentiated into a neutrophilic phenotype, and that can be transfected efficiently and reliably, would prove a most useful research tool to extend our understanding of cytokine generation and upstream processes in human granulocytes.

Various approaches have already been employed to differentiate the human pro-myeloid cell line PLB-985 into neutrophil-like cells [10-17]. However, the functional properties of granulocytic PLB-985 cells have only been defined for some cellular responses, such as degranulation and the associated respiratory burst [17], or arachidonc acid metabolism [14]. Thus, much remains to be determined concerning the suitability of differentiated PLB-985 to serve as a cellular model for primary neutrophils, especially in the case of more recently described neutrophil functional responses, such as the production of inflammatory cytokines and chemokines, and the underlying activation of discrete transcription factor families. This being said, PLB-985 cells represent a potentially attractive model since they are transfectable. Indeed, a few groups have stably transfected PLB-985 cells prior to granulocytic differentiation for specific purposes [14,18-20]. However, stable transfections are not always feasible, as the overexpressed proteins can interfere with granulocytic differentiation, a process which typically spans 4 to 6 days. Transient transfection of PLB-985 cells, on the other hand, has only been achieved in isolated instances, and with mitigated success [16,21]. This owes much to drawbacks inherent to the transfection techniques used, i.e. electroporation and cationic liposome-mediated transfection. Indeed, electroporation of PLB-985 cells reportedly results in high mortality rates while only achieving moderate transfection efficiency [16,21], whereas the liposome-based approach had a low transfection efficiency and required that the cells be pretreated with TPA for 4 h prior to transfection [21]. This in itself is problematic, because TPA is a differentiating agent that commits the cells to the monocytic lineage [10]. In contrast, a recent paper, which was published while we were completing the current study, reported the efficient transfection of PLB-985 cells with the nucleofection technique [22], an approach which entails lower mortality rates.

In the present study, we ascertained that granulocytic PLB-985 cells respond like primary neutrophils in terms of inflammatory cytokine production and transcription factor activation. We then optimized the transient transfection of granulocytic PLB-985 cells using the nucleofection technique, which yields high transfection efficiencies, relatively low mortality rates, and rapid overexpression of various proteins. We also show that this approach lends itself well to promoter activation studies in neutrophil-like PLB-985 cells. We finally applied this approach to confirm the implication of NF-κB in inflammatory cytokine production by human granulocytes, and to elucidate the long-standing issue of whether endogenous reactive oxygen intermediates (ROI)^1 ^influence transcription factor activation and downstream processes in cells that are heavy ROI producers, such as neutrophilic granulocytes.

## Results

### Granulocytic differentiation of PLB-985 cells

**Figure 1:**
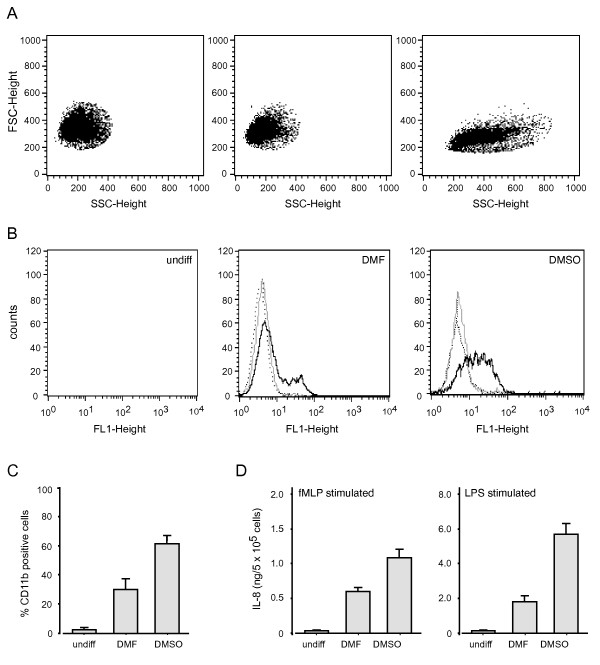
**Acquisition of granulocytic features following differentiation of PLB-985 cells.** (A) Cells were differentiated for 5 days in the presence of DMF or DMSO, or left undifferentiated, prior to FACS determination of their size (forward scatter) and granularity (side-scatter). (B) Cells were differentiated for 5 days in the presence of DMF or DMSO, or left undifferentiated, prior to staining with an anti-CD11b Ab (black trace), with an isotype-matched control Ab (grey trace) or without a first Ab (dotted trace) and subsequent incubation with a FITC-conjugated 2nd Ab. CD11b surface expression was then determined by FACS analysis; a minimum of 10,000 cells were processed for each sample. Results are representative of at least five independent experiments. (C) Results obtained as described above were compiled and expressed as mean ± s.e.m. of at least 5 independent experiments. For comparison, 100% of human neutrophils express CD11b on their surface. (D) Cells were differentiated for 5 days in the presence of DMF or DMSO, or left undifferentiated, prior to stimulation for 6 h at 37°C with fMLP or LPS. Culture supernatants were then collected and analyzed by ELISA. Mean ± s.e.m. of at least 6 independent experiments.

We initially differentiated PLB-985 cells using either DMSO or DMF, as both agents were reported to promote granulocytic differentiation [10-13,15,17]. Culture of PLB-985 cells with either agent resulted in a gradual inhibition of proliferation (that was most pronounced with DMSO). By day 5 of differentiation, over 80% of the cells had acquired a typically neutrophilic morphology i.e. lobular nuclei, and all of the cells had an increased granularity (Figure 1A), as reported previously [16,17]. Differentiating PLB-985 cells also displayed an augmented expression of cell surface markers such as the integrin, CD11b – a phenomenon that was especially evident in DMSO-differentiated cells (Figure 1B and 1C). Similarly, neutrophil-like PLB-985 cells acquired the ability to generate IL-8 in response to LPS and to the chemoattractant, fMLP (Figure 1D), in keeping with the observation that granulocytic differentiation correlates with the onset of CD14 and fMLP receptor surface expression [17,23]. Again, the acquisition of fMLP and LPS responsiveness was most marked in DMSO-differentiated cells (Figure 1D). Together, the above observations indicate that DMSO treatment leads to a more complete differentiation of PLB-985 cells into a neutrophil-like phenotype. As a result, we utilized DMSO as a differentiating agent in all subsequent experiments.

### Cytokine expression profile of granulocytic PLB-985 cells

**Figure 2:**
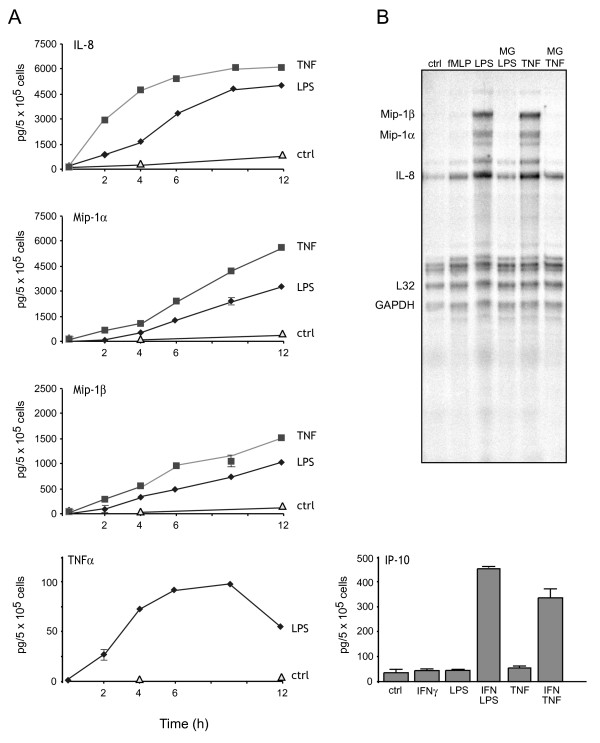
**Cytokine expression profile of neutrophil-like PLB-985 cells.** (A) DMSO-differentiated PLB-985 cells (day 5) were cultured in the absence ("ctrl") or presence of either 100 ng/ml LPS or 100 U/ml TNFα for the indicated times. When IP-10 was measured, the cells were instead cultured for 9 h with the above stimuli, in the presence or absence of 100 U/ml IFNγ. Culture supernatants were then harvested and their cytokine content determined in ELISA. Results are representative of at least 4 independent experiments. (B) DMSO-differentiated PLB-985 cells (day 5) were pre-treated with or without MG-132 (15 μM, 30 min, 37°C), and stimulated in the absence ("ctrl") or presence of either 30 nM fMLP, 100 ng/ml LPS or 100 U/ml TNFα for 30 min at 37°C. Total RNA was extracted and cytokine mRNA expression was analyzed in RPA. Results shown in this figure are representative of at least 4 independent experiments.

We next determined the extent to which the cytokine expression pattern of DMSO-differentiated PLB-985 cells conforms to that of primary neutrophils. To this end, granulocytic PLB-985 cells were stimulated with either LPS or TNF for increasing lengths of time, and cytokine release was measured. As shown in Figure 2A, neutrophil-like PLB-985 cells generate IL-8, Mip-1α, Mip-1β, and TNFα with kinetics that are very reminiscent of those observed in human neutrophils [24-26]. Another similarity between granulocytic PLB-985 cells and human neutrophils is the delayed IL-8 production kinetics observed in response to LPS (compared to those elicited by TNF), which reflects a requirement for endogenous TNFα in LPS-treated neutrophils [27]. Accordingly, stimulation of granulocytic PLB-985 cells with LPS in the presence of a neutralizing anti-TNF antibody reduced IL-8 secretion by 48 ± 5 % (mean ± s.d., n = 2) at the 12-h time point. More strikingly, the generation of IP-10 by granulocytic PLB-985 cells (Figure 2A) shares the same singular induction characteristics which we originally reported for primary neutrophils [28], i.e. the requirement for a co-stimulation using IFNγ with either LPS or TNFα. When the expression of the corresponding cytokine genes was investigated, LPS and TNF were found to be good inducers in differentiated PLB-985 cells (Figure 2B and data not shown), again in keeping with the induction characteristics of the same cytokine and chemokine genes observed in human neutrophils. Thus, it appears that DMSO-differentiated PLB-985 cells have a resolutely neutrophilic cytokine expression profile.

### Transcription factor binding profile of granulocytic PLB-985 cells

**Figure 3:**
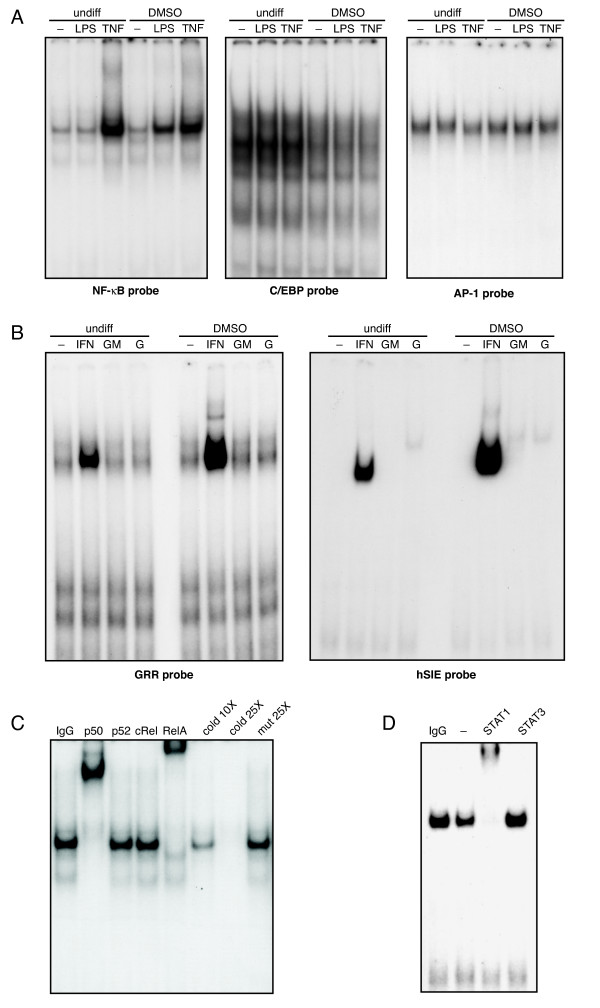
**Transcription factor binding profile of undifferentiated and neutrophil-like PLB-985 cells.** (A) Undifferentiated or DMSO-differentiated PLB-985 cells (day 5) were incubated for 15 min at 37°C in the absence ("-") or presence of 100 ng/ml LPS or 100 U/ml TNFα, and nuclear extracts were prepared and analyzed in EMSA (1 μg/lane) using NF-κB, C/EBP, or AP-1 oligonucleotide probes. (B) Undifferentiated or DMSO-differentiated PLB-985 cells (day 5) were incubated for 15 min at 37°C in the absence ("-") or presence of 100 U/ml IFNγ, 1 nM GM-CSF, or 1000 U/ml G-CSF, and nuclear extracts were prepared and analyzed in EMSA (1 μg/lane) using GRR or hSIE oligonucleotide probes. (C) Nuclear extracts from TNF-stimulated granulocytic PLB-985 cells (day 5) were incubated for 30 min in binding buffer in the presence of antibodies to p50, p52, c-Rel, RelA, or an isotype-matched antibody ("IgG"), or in the presence of a 10- or 25-fold molar excess of unlabeled NF-κB oligonucleotide probe ("cold"), or in the presence of a 25-fold molar excess of unlabeled NF-κB probe featuring a mutated NF-κB site ("mut"), prior to the addition of labeled NF-κB probe and subsequent EMSA analysis. (D) Nuclear extracts from IFNγ-stimulated granulocytic PLB-985 cells (day 5) were incubated for 30 min in binding buffer in the absence ("-") or presence of antibodies to STAT1, STAT3, or an isotype-matched antibody ("IgG"), prior to the addition of labeled hSIE/m67 probe and subsequent EMSA analysis. Experiments shown in this figure are representative of at least three.

The induction of inflammatory cytokines is to a large extent under the control of discrete transcription factors (mainly from the NF-κB, C/EBP, AP-1, and STAT families), and we indeed observed that the expression of most cytokines investigated in granulocytic PLB-985 cells can be inhibited by MG-132 (Figure 2B and data not shown), a proteasome inhibitor which prevents the proteasome-dependent degradation of IκB-α and subsequent NF-κB activation [29,30]. This led us to characterize the binding of the aforementioned transcription factors in this cellular model. As shown in Figure 3A, neither LPS nor TNFα could increase the binding of AP-1 or C/EBP complexes over constitutive levels, be it in granulocytic or undifferentiated PLB-985 cells. By comparison, TNFα potently activated NF-κB independently of the PLB-985 differentiation stage, and LPS also proved to be a strong inducer in granulocytic PLB-985 cells (Figure 3A, left panel). The inability of LPS to activate NF-κB in undifferentiated PLB-985 cells (Figure 3A, left panel) is consistent with the lack of detectable CD14 in these cells [23]. The major inducible NF-κB DNA-binding complex of granulocytic PLB-985 cells was found to be specific, since it was competed out by a 25-fold excess of unlabeled probe in the binding mix, but unaffected by a 25-fold excess of unlabeled oligonucleotide in which the NF-κB sequence was mutated (Figure 3C, last 2 lanes). To identify the constituents of this specific NF-κB complex, supershift assays were performed. As shown in Figure 3C, the inducible NF-κB band was efficiently supershifted by antibodies against p50 and RelA, suggesting that it represents the prototypical p50/RelA homodimer, as in primary human neutrophils [31].

We also investigated inducible STAT binding in response to stimuli which potently induce this response in neutrophils. For this purpose, we used two different oligonucleotide probes. The first is the gamma response region (GRR) of the CD64 gene, which can detect binding of STAT1-, STAT3- and STAT5-containing complexes [32]. The other probe is the human serum-inducible element (hSIE) found within the c-*fos *promoter, and which binds STAT1- and STAT3-containing complexes with very high affinity [33]. As shown in Figure 3B, IFNγ proved to be a good inducer of STAT binding in undifferentiated PLB-985 cells, and even more so in granulocytic PLB-985 cells. By comparison, G-CSF and GM-CSF weakly induced a hSIE-binding activity (Figure 3B, right panel). In the particular case of GM-CSF, it is noteworthy that this inducible activity was only observed in neutrophil-like PLB-985 cells, consistent with the observation that the GM-CSF receptor is barely expressed in undifferentiated PLB-985 cells and increases considerably during granulocytic differentiation [17]. The identity of the major inducible STAT-containing complex (i.e. that observed in IFNγ-stimulated cells) was finally investigated in supershift assays. Antibodies against STAT1 completely supershifted the hSIE complex, whereas antibodies directed against other STAT isoforms were without effect (Figure 3D and data not shown), suggesting that the inducible complex represents STAT1 homodimers, as we initially reported in primary human neutrophils [34,35].

Together, the transcription factor binding characteristics of granulocytic PLB-985 cells closely match those observed in human neutrophils [31,34,36]. Moreover, supershift analyses showed that the inducible NF-κB and STAT complexes of granulocytic PLB-985 cells have the same subunit composition as those of primary neutrophils, i.e. p50/RelA heterodimers in the case of the NF-κB complex, STAT1 homodimers in the case of IFNγ-induced complexes (Figure 3C, 3D).

### Transient overexpression of proteins in granulocytic PLB-985 cells

**Figure 4:**
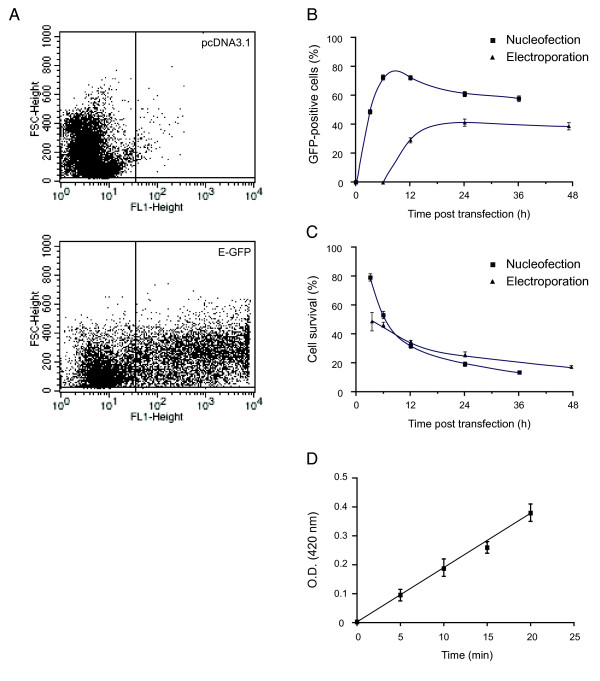
**Overexpression of E-GFP and β-galactosidase in neutrophil-like PLB-985 cells.** (A) DMSO-differentiated PLB-985 (day 5) were nucleofected with a vector encoding E-GFP or empty vector (pcDNA3.1). Cells were harvested 6 h after nucleofection and processed for FACS analysis. A minimum of 10,000 cells was analyzed per sample. Results are representative of 8 independent experiments. (B) DMSO-differentiated PLB-985 (day 5) were nucleofected (squares) or electroporated (triangles) with an E-GFP vector and cultured for the indicated times prior to propidium iodide staining and FACS analysis. Results are expressed as the proportion of GFP-expressing cells in the population that excludes PI staining. Mean ± s.e.m. of 5 independent experiments. (C) DMSO-differentiated PLB-985 (day 5) were nucleofected (squares) or electroporated (triangles) with an E-GFP vector and cultured for the indicated times prior to determination of cell survival. Mean ± s.e.m. of 5 independent experiments. (D) DMSO-differentiated PLB-985 (day 5) were nucleofected with a β-galactosidase vector and cultured for 6 h. Cells were then lysed and processed for their enzymatic activity, using 1-*O*-[2-nitrophenyl]-β-*D*-galactopyranoside as a substrate. Results are of two independent experiments.

Having ascertained that granulocytic PLB-985 cells represent a suitable cellular model for the study of inflammatory cytokine generation in primary neutrophils, we turned our attention to the introduction of constructs into these cells. Although isolated studies have shown that it is possible to transiently transfect PLB-985 cells using electroporation, transfection efficiency is reportedly modest, and the mortality rates are very high [16,21]. We obtained similar results when we introduced pcDNA3.1 vector containing a full-length E-GFP insert granulocytic PLB-985 by electroporation under a variety of settings. We subsequently used the nucleofection technique, which proved far superior to electroporation in a number of ways. As shown in Figure 4A, granulocytic PLB-985 cells nucleofected with the same E-GFP vector consistenly displayed a large increase in fluorescence, relative to cells nucleofected with vector alone. In 8 independent experiments, transfection efficiency was 73 ± 4% at 6 h postnucleofection, which proved to be the optimal time (Figure 4B). By comparison, electroporation of the same vector into granulocytic PLB-985 cells required much longer to reach an optimal expression efficiency that was lower than the one obtained by nucleofection (Figure 4B). Like electroporation, nucleofection is known to cause irreversible damages to a fraction of the cell population, even under optimized conditions. As shown in Figure 4C, the survival rate of granulocytic PLB-985 cells following nucleofection was 56 ± 4% at 6 h (n = 8) and gradually decreased to about 20% 24 h. By comparison, electroporation of the same vector resulted in much higher initial mortality rates (Figure 4C). Thus, nucleofection proved to be a rapid, reliable, efficient, and reasonably gentle method of overexpressing a protein into granulocytic PLB-985 cells.

**Figure 5:**
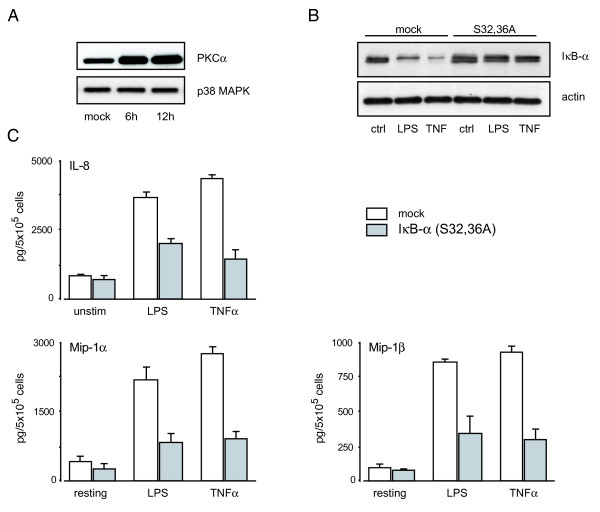
**Transient overexpression of proteins in neutrophil-like PLB-985 cells.** (A) DMSO-differentiated PLB-985 (day 5) were nucleofected with a plasmid encoding human PKCα or with vector alone (lane 1). The cells were cultured for 6 or 12 h after nucleofection, and processed for immunoblot analysis of their PKCα content. Membranes were then reblotted with a p38 MAPK antibody (as a loading control). (B) DMSO-differentiated PLB-985 (day 5) were nucleofected with a plasmid encoding a dominant negative form of human IκB-α (S32,36A), or with empty vector ("mock"). The cells were cultured for 6 h after nucleofection, and stimulated for 45 min with 100 ng/ml LPS or 100 U/ml TNFα, prior to immunoblot determination of their IκB-α or actin content. (C) DMSO-differentiated PLB-985 (day 5) were nucleofected with a plasmid encoding human IκB-α (S32,36A), or with empty vector ("mock"). The cells were cultured for 6 h after nucleofection, and stimulated for another 6 h in the absence ("resting") or presence of 100 ng/ml LPS or 100 U/ml TNFα. Culture supernatants were then harvested and analyzed in ELISA for the indicated cytokines. Results shown in this figure are representative of three independent experiments.

Having successfully introduced E-GFP into granulocytic PLB-985 cells, we next determined whether our protocol could be used for the transient overexpression of proteins that feature an enzymatic activity. For this purpose, the cells were nucleofected with a plasmid encoding β-galactosidase. As shown in Figure 4D, a β-galactosidase activity was readily detectable in transfected granulocytic PLB-985 cells. We next attempted to transfect a more physiologically relevant protein. For this purpose, we used a plasmid encoding wild type PKCα. As shown in Figure 5A, granulocytic PLB-985 cells had largely increased PKCα cellular levels 6 h after nucleofection. When we examined the effect of overexpressing PKCα on the cytokine expression profile of PLB-985 cells, however, no significant change was noted relative to mock-transfected cells (data not shown). We also overexpressed a dominant negative IκB-α mutant (S32,36A) in neutrophil-like PLB-985 cells. Although this did not result in any apparent change in the overall expression of IκB-α, cells transfected with the dominant negative mutant were rendered resistant to inducible IκB-α degradation (Figure 5B). Another functional consequence of introducing the S32,36A mutant was a profound inhibition of inflammatory cytokine production by granulocytic PLB-985 cells (Figure 5C), which confirms that these cytokines are under the control of NF-κB, as observed in primary neutrophils [37].

### Transient transfection of granulocytic PLB-985 cells with promoter-reporter constructs

**Figure 6:**
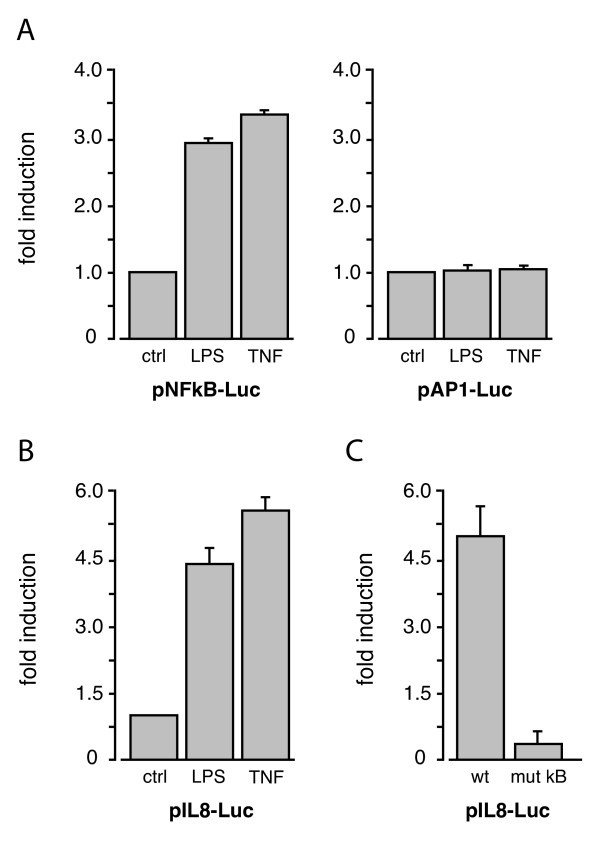
**Transient transfection of neutrophil-like PLB-985 cells with promoter-reporter constructs.** (A) Granulocytic PLB-985 cells (day 5) were nucleofected with luciferase constructs driven by 5 repeated NF-κB elements ("pNFkB-Luc"), or by 7 repeats of the consensus AP-1 element ("pAP1-Luc"). The cells were then cultured for 6 h at 37°C in the presence or absence of 100 ng/ml LPS or 100 U/ml TNFα, prior to cell lysis and luciferase activity measurement. Mean ± s.e.m. of at least 3 independent experiments. Transfections made using empty vectors yielded values that did not differ significantly from background, whether in resting or stimulated cells (not shown). (B) Granulocytic PLB-985 cells (day 5) were nucleofected with a luciferase construct driven by the IL-8 promoter ("pIL-8-Luc"), and cultured for 6 h at 37°C in the presence or absence of 100 ng/ml LPS or 100 U/ml TNFα, prior to cell lysis and luciferase activity measurement. Mean ± s.e.m. from 8 independent experiments. (C) Granulocytic PLB-985 cells (day 5) were nucleofected with luciferase constructs encoding either either a wild-type ("wt") IL-8 promoter or a variant mutated in the NF-κB site ("mut κB"). The cells were then cultured for 6 h at 37°C in the presence or absence of 100 U/ml TNFα, prior to cell lysis and luciferase activity measurement. Mean ± s.e.m. from 2 independent experiments.

We next investigated whether it would be possible to carry out promoter studies in granulocytic PLB-985 cells. For this purpose, the cells were nucleofected with luciferase constructs under the control of NF-κB or AP-1 enhancer elements. As shown in Figure 6A, cell stimulation with either LPS or TNFα resulted in a marked induction of NFκB-driven promoter activity, whereas a no significant induction was observed with the AP-1-driven construct. These results prompted us to investigate the ability of differentiated PLB-985 cells to activate an actual chemokine promoter. To this end, the cells were nucleofected with a construct containing the IL-8 promoter coupled to a luciferase reporter gene. As shown in Figure 6B, both LPS and TNF stimulation led to a robust activation of the IL-8 promoter. These results agree well with data from primary neutrophils, in which IL-8 gene transcription and NF-κB (but not AP-1) are potently activated in response to LPS or TNFα [31,36,38,39]. We finally sought to confirm the impact of NF-κB on IL-8 promoter activation. To this end, granulocytic PLB-985 cells were transfected with luciferase constructs featuring either a wild-type proximal IL-8 promoter, or a variant mutated within the κB site. As shown in Figure 6C, the mutant IL-8 promoter was mostly resistant to stimulation with TNFα; similar results were obtained in LPS-stimulated cells (not shown). This is consistent with the data presented in Figure 5C, and is highly reminiscent of the essential role played by NF-κB in IL-8 gene induction in neutrophils [36].

### Role of endogenous reactive oxygen intermediates (ROI) in transcription factor activation and downstream processes in human granulocytes

**Figure 7:**
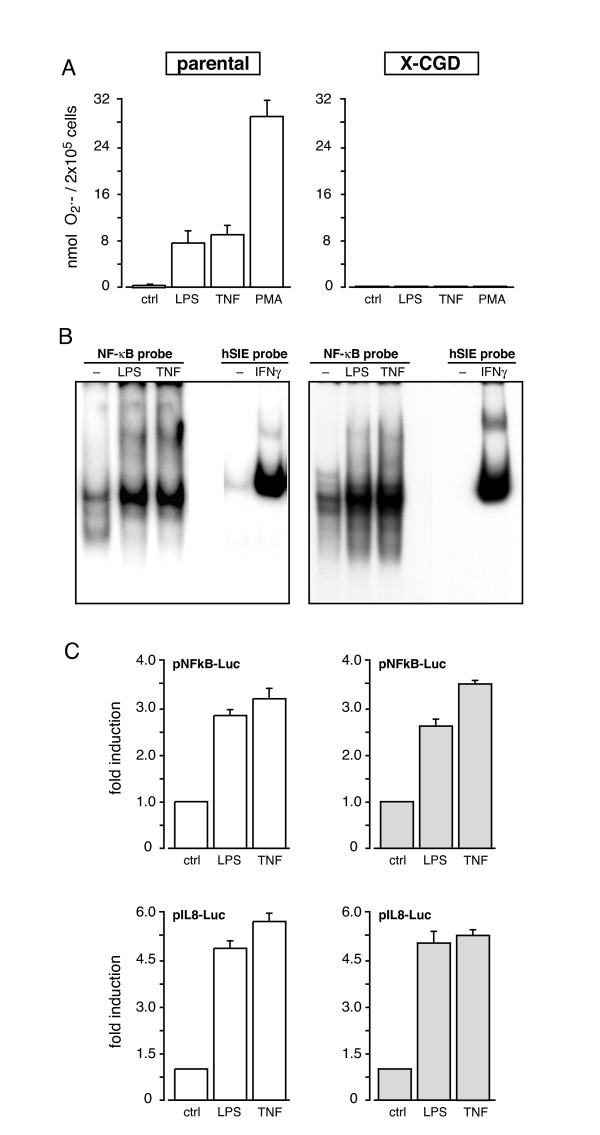
**Responsiveness of neutrophil-like X-CGD PLB-985 cells and their parental counterparts.** (A) Granulocytic (day 5) X-CGD PLB-985 cells (grey bars) or parental controls (open bars) were incubated for 30 min in the presence or absence of 100 ng/ml LPS, 100 U/ml TNFα, or 25 nM PMA (as a positive control), prior to the determination of superoxide generation by the cytochrome c reduction assay. Mean ± s.e.m. from at least 3 independent experiments. (B) Granulocytic (day 5) X-CGD PLB-985 cells (right panel) or parental controls (left panel) were incubated for 15 min at 37°C in the absence ("-") or presence of 100 ng/ml LPS, 100 U/ml TNFα, or 100 U/ml IFNγ, and nuclear extracts were prepared and analyzed in EMSA using a NF-κB or hSIE oligonucleotide probe. This experiment is representative of three. (C) Granulocytic (day 5) X-CGD PLB-985 cells (grey bars) or parental controls (open bars) were nucleofected with luciferase constructs driven by 5 repeated NF-κB elements ("pNFκB-Luc"), or by the IL-8 promoter ("pIL-8-Luc"), and cultured for 6 h at 37°C in the presence or absence of 100 ng/ml LPS or 100 U/ml TNFα, prior to cell lysis and luciferase activity measurement. Mean ± s.e.m. from 3 independent experiments.

Endogenously generated ROI have been implicated in the regulation of NF-κB activation in several cellular models (reviewed in [40,41]). In neutrophils, however, the role of endogenous ROI in transcription factor activation and cytokine generation remains unclear, despite the fact that neutrophils probably produce more ROI than any other cell type. To address this issue, we used X-CGD PLB-985 cells, in which the NADPH oxidase is inactive due to the targeted disruption of a key component, gp91phox [42]. Indeed, Figure 7A shows that whereas granulocytic PLB-985 cells respond to TNFα, LPS, or to more classical NADPH oxidase activators (such as PMA), granulocytic X-CGD cells completely lack this ability, in agreement with previous studies [19,42,43]. We next investigated the ability of LPS and TNFα to activate NF-κB in DMSO-differentiated X-CGD PLB-985 cells. We additionally examined the ability of IFNγ to induce STAT1 binding to DNA in these cells, since this process can also be affected by endogenous reactive oxygen species in some instances [44,45]. As shown in Figure 7B, NF-κB was robustly activated by LPS and TNFα, to an extent comparable to that observed in wild-type PLB-985 cells (Figure 3A). Similarly, STAT1 binding to hSIE in response to IFNγ was potently induced, both in X-CGD granulocytic PLB-985 cells (Figure 7B) and in their wild-type counterparts (Figure 3A). Thus, activation of these two key transcription factors does not appear to be influenced by the production of endogenous ROI in human granulocytes. We next investigated whether the transcriptional activity of promoter constructs might nevertheless be affected in DMSO-differentiated X-CGD PLB-985 cells. To this end, the cells were transfected with either pNFκB-Luc or pIL8-Luc, and stimulated with LPS or TNFα. As shown in Figure 7C, each construct was activated to a similar extent in X-CGD granulocytes as in the parental cell line, in agreement with our NF-κB binding data in wild-type vs X-CGD granulocytic PLB-985 cells. Finally, we examined the release of three κB-dependent chemokines (IL-8, Mip-1α, Mip-1β) in DMSO-differentiated X-CGD PLB-985 cells stimulated with LPS or TNFα. Expectedly, all three chemokines were released with similar amplitude and kinetics as in wild-type PLB-985 cells (data not shown). Thus, NF-κB activation and dowstream responses do not appear to be under the control of endogenous ROI in human granulocytes.

**Figure 8:**
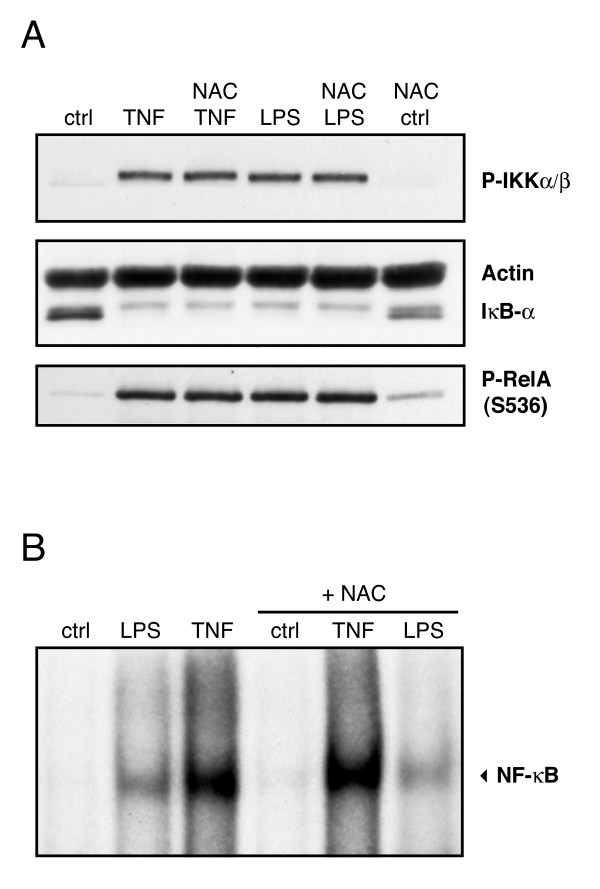
**Effect of N-acetyl cysteine on the IKK/NF-κB pathway in human neutrophils.** (A) Neutrophils were pretreated with culture medium containing 20 mM N-acetyl cysteine ("NAC") or diluent control for 30 min at 37°C, and further incubated in the absence ("ctrl") or presence of 100 ng/ml LPS or 100 U/ml TNFα. Samples were then processed for SDS-PAGE and immunoblot analysis. Note: actin and IκB are shown on the same blot, as the membrane was first immunoblotted for IκB-α, and then re-blotted for actin. (B) Neutrophils were pretreated with N-acetyl cysteine or diluent as described above, and further incubated in the absence or presence of 100 ng/ml LPS or 100 U/ml TNFα. Nuclear extracts were then prepared and analyzed in EMSA using a NF-κB oligonucleotide probe. The experiments depicted are representative of at least two.

To ascertain whether these results properly reflect the behavior of primary neutrophils, we cultured the latter cells in the presence of the antioxidant, N-acetyl cysteine, prior to stimulation with LPS or TNFα, and assessment of the IKK cascade activation. As shown in Figure 8, N-acetyl cysteine pre-incubation failed to affect the inducible phosphorylation of IKKα/β and RelA, or the inducible degradation of IκB-α, in human neutrophils. Accordingly, the inducible DNA binding of NF-κB was also unaffected under the same conditions (Figure 8B). Identical results were obtained when neutrophils were pretreated with a different antioxidant, PDTC, as reported before [46,37]. By comparison, N-acetyl cysteine pretreatment abolished superoxide generation from fMLP- or PMA-stimulated neutorphils (data not shown). Together, these results strongly suggest that NF-κB activation and κB-dependent responses (such as inflammatory cytokine generation) are independent of endogenous ROI in human neutrophils.

## Discussion

In this study, we characterized a transfectable cellular model whose behavior closely corresponds to that of primary human neutrophils, both phenotypically and functionally. Indeed, PLB-985 cells that had been differentiated along the granulocytic lineage displayed neutrophil-like surface markers, and became responsive to physiological neutrophil stimuli such as fMLP, LPS, and GM-CSF. More importantly, transcription factor activation profile and cytokine expression kinetics of granulocytic PLB-985 cells were found to faithfully match those observed in primary neutrophils. Perhaps most strikingly, the generation of IP-10 by granulocytic PLB-985 cells shares the same singular induction characteristics that we originally reported for primary neutrophils [28], i.e. the requirement for a co-stimulation using IFNγ with either LPS or TNFα. Whereas neutrophils are notoriously refractory to transfection, this limitation was overcome in granulocytic PLB-985 cells, thereby making it possible to study promoter activation, as well as the impact of overexpressed proteins on cytokine production and related events. This allowed us to confirm the central role of NF-κB in the production of cytokines by granulocytes, and to show that κB-dependent responses are independent of endogenous reactive oxygen intermediates in these cells.

From a technical perspective, our data shows that the nucleofection approach (as opposed to electroporation or lipofection) is particularly well suited for the transient overexpression of various proteins into PLB-985 granulocytes. Indeed, the high transfection efficiency and relatively moderate mortality rates allowed us to successfully introduce E-GFP, β-galactosidase, wt PKCα, and dn IκB-α into these cells. As this study was nearing completion, Boulven *et al*. similarly overexpressed two mutant PI 3-kinase subunits in differentiated PLB-985 cells using a nucleofection approach similar to the one described herein [22]. Although their nucleofector settings and nucleofection buffers were different than those used herein, they yielded comparable transfection efficiencies (T. Ear, unpublished data), thereby further validating the nucleofection approach. From a more functional perspective, our overexpression of dn IκB-α prevented its degradation in response to physiological stimuli, thereby severely impairing the generation of several inflammatory cytokines that are known to be under the control of NF-κB. The fact that dn IκB-α overexpression yielded a greater than 50% inhibition of IL-8, Mip-1α and Mip-1β production is especially relevant, considering that the E-GFP overexpression experiments indicated that about 70% of the cells actually express the transfected material. Thus, dn IκB-α must have been very effective in inhibiting downstream cellular responses in those cells which overexpressed the mutant protein. At any rate, these results confirm the pivotal role of NF-κB in the inducible generation of inflammatory cytokines in human granulocytes, in keeping with our previous observations made in primary neutrophils using various pharmacological inhibitors of the NF-κB pathway [37,47]. Collectively, our overexpression experiments foreshadow exciting new advances in our understanding of neutrophil biology, insofar as they make possible the future dissection of the various signal transduction pathways controlling cytokine production and transcription factor activation in human granulocytes.

The outcome of the experiments in which we introduced luciferase contructs into granulocytic PLB-985 cells also confirmed and extended several observations made in primary neutrophils. In particular, the inducibility of NF-κB-driven luciferase constructs (but not of AP1-driven constructs) agrees well with the observation that NF-κB can be activated in neutrophils stimulated with LPS or TNFα [31,34], whereas AP-1 cannot [36,39]. In this regard, it is also noteworthy that the activation of the NF-κB-driven luciferase construct represents the first direct demonstration of the ability of a transcription factor to transactivate a downstream gene in human granulocytes. A more compelling example is our demonstration that LPS and TNFα promote the transactivation of the IL-8 promoter in granulocytic PLB-985 cells, as the IL-8 gene is known to be under the control of NF-κB in human neutrophils [37]. Accordingly, we showed that a proximal IL-8 promoter mutated within its κB site became mostly unresponsive to stimulation. Again, it is worth noting that the activation of the IL-8 promoter in PLB-985 granulocytes confirms our previous observations made in primary neutrophils, which had shown that the IL-8 gene can be transcriptionally activated by the same stimuli, as determined in nuclear run-on analyses [38] and primary transcript PCR [47]. From a more general standpoint, the above considerations establish that the nucleofection of PLB-985 granulocytes described herein paves the way for detailed promoter studies in human granulocytes – an enterprise which had heretofore remained elusive.

In a variation of the aforementioned promoter studies, we applied the approach described herein to elucidate the long-standing issue of whether endogenously generated ROI contribute to transcription factor activation and cytokine production in granulocytes, as already reported for several other cellular models [40,41]. For this purpose, we used DMSO-differentiated X-CGD PLB-985 cells, in which the ROI-generating NADPH oxidase complex is inactive. Following LPS or TNF stimulation, these X-CGD PLB-985 cells were found to behave in strikingly similar fashion to their wild-type counterparts, be it in terms of promoter activation (using pNFκB-Luc or pIL8-Luc), transcription factor activation (NF-κB, STAT) and inflammatory cytokine generation (IL-8, Mip-1α, Mip-1β). These results strongly indicate that NF-κB and STAT activation, as well as dowstream responses, are independendent of endogenous ROI in human granulocytes. This conclusion is further supported by experiments made in primary neutrophils, in which a powerful antioxidant (N-acetyl cysteine) similarly failed to affect the activation of the IKK/IκB-α/NF-κB cascade [46]. This being said, it could be argued that since LPS and TNF are not known as strong NADPH oxidase activators, then it stands to reason that endogenous ROI should play little or no role in cellular processes. In this regard, Selmeczy and colleagues reported that TNF secretion in response to opsonized zymosan was nearly abolished in granulocytic (DMF-differentiated) X-CGD cells, compared to parental controls [48]. However, this was attributable to the much lower levels of surface CD16 found in these X-CGD granulocytes [48]. On the opposite, ROI are abundantly produced in phagocytozing neutrophils, and it was reported that NF-κB activation under these conditions was unchanged in the presence of various oxidant scavengers (exogenous catalase, superoxide dismutase, or methionine), and that conversely, exogenous H_2_O_2 _failed to activate NF-κB [49]. In agreement with these results, we also observed that exogenous H_2_O_2 _(up to 1 mM) does not induce NF-κB or STAT activation in nonphagocytozing neutrophils (our unpublished data). It is perhaps precisely because neutrophils are such prolific producers of ROI that they are so well protected from their adverse effects. In this regard, the specific activity of catalase was described to be at least 4-fold higher in neutrophils vs all other phagocytes, and neutrophil function was reportedly unaffected by 0.5 mM exogenous H_2_O_2 _over several hours [50]. Similarly, it was found that among human blood cells, neutrophils uniquely express high levels of methionine-sulfoxide-reductase enzymes [51]. Whatever the case may be, it has now become quite clear that neither endogenous ROI, nor exogenously provided ROI (such as H_2_O_2_) significantly affect NF-κB activation and dowstream processes (such as inflammatory cytokine production) in human neutrophils. Studies are now under way to further decipher the intricacies of transcription factor regulation in human granulocytes.

## Conclusion

In this report, we characterized a transfectable cellular model whose behavior closely corresponds to that of primary human neutrophils, both phenotypically and functionally, with a particular emphasis on a prominent functional response of these cells, i.e. inflammatory cytokine production, as well as some key underlying processes such as transcription factor activation. Using this model, we confirmed the pivotal role of NF-κB in the onset of cytokine production, and further showed that NF-κB activation is independent of endogenous oxygen-derived intermediates. Because such studies were heretofore impossible to carry out in primary human neutrophils, the approach which we describe is likely to enable significant advances in our understanding of various aspects of neutrophil biology.

## Methods

### Antibodies and reagents

Antibodies raised against NF-κB/Rel proteins, IκB-α, C/EBP isoforms, PKCα, and actin were from Santa Cruz Biotechnology (Santa Cruz, CA, USA), and the anti-CD11b was from BD Biosciences (Mississauga, Canada). Ficoll-Paque, T4 polynucleotide kinase and poly (dI-dC) were from Amersham-Pharmacia (Baie d'Urfé, Qc, Canada); radionucleotides were from NEN (Boston, MA, USA). Endotoxin-free (< 2 pg/ml) RPMI 1640 and FCS were from Sigma (St-Louis, MO, USA) and Wisent (St-Bruno, Qc, Canada), respectively. Recombinant human cytokines were from R&D Systems (Minneapolis, MN, USA), and UltraPure LPS (from *E. coli *0111:B4) was from InvivoGen (San Diego, CA, USA). Acetylated BSA, diisopropyl fluorophosphate (DFP), dimethyl formamide (DMF), dimethyl sulfoxide (DMSO), N-formyl-methionyl-phenylalanine (fMLP), and phenylmethanesulphonyl fluoride (PMSF) were from Sigma-Aldrich (St. Louis, MO, USA). Aprotinin, 4-(2-aminomethyl)benzenesulfonyl fluoride (AEBSF), leupeptin, Nutridoma-SP, and pepstatin A were from Roche (Laval, Qc, Canada). All other reagents were of the highest available grade, and all buffers and solutions were prepared using pyrogen-free clinical grade water.

### Plasmids

A plasmid encoding β-galactosidase in the pCMVβ vector was from Clontech (Mountain View, CA, USA). Plasmids containing luciferase contructs under the control of 5 repeated NF-κB elements (pNFκB-Luc) or 7 repeated AP-1 elements (pAP1-Luc) were from Stratagene (La Jolla, CA, USA). Plasmids containing luceferase contructs encoding the full IL-8 promoter (-1498 to +44), a proximal IL-8 promoter (-162 to +44), or a version of the latter mutated within its κB site [52], were a kind gift from Dr. Allan R. Brasier (University of Texas Medical Branch). A construct encoding a dominant negative form of IκB-α (S32/36A) was obtained from Dr. Christian Jobin (University of North Carolina at Chapel Hill), and a construct encoding wild type PKCα was from Dr. Gilles Dupuis (Université de Sherbrooke); both constructs were subcloned into pcDNA3.1. Similarly, cDNA sequences corresponding to GFP (ex 488 nm, em 507 nm) or β-galactosidase were respectively excised from the commercial vectors, pEGFP-N1 and pCMVb (Clontech), and subcloned into pcDNA3.1. Following amplification in DH-5α bacteria, all plasmids were purified using Maxiprep kits featuring EndoFree buffers for endotoxin removal (Qiagen).

### Cell isolation and culture

Neutrophils were isolated from the peripheral blood of healthy donors as described previously [46]; each blood donor gave informed consent under a protocol that had been duly approved by the ethics committee of our Research center (comité d'éthique humaine du Centre de recherche du CHUS). As determined by Wright staining and nonspecific esterase cytochemistry, the final neutrophil suspensions consistently contained fewer than 0.5% mononuclear cells; neutrophil viability exceeded 98% after up to 4 h in culture, as determined by trypan blue exclusion. The myelomonoblastic PLB-985 cell line was purchased from the Deutsche Sammlung von Mikroorganismen und Zellkulturen GmbH (Braunschweig, Germany). X-CGD PLB-985 cells, which feature a targeted disruption of the gene encoding the essential NAPDPH subunit, gp91phox [42], were a kind gift from Dr. Mary Dinauer (Indiana University, Indianapolis, IN). All cells were cultured at 37°C under a humidified 5% CO_2 _atmosphere in RPMI 1640 containing 10% FCS, 100 U/ml penicillin and 100 μg/ml streptomycin (hereafter referred to as complete RPMI medium). To induce granulocytic differentiation, 1.25% DMSO was added to the culture medium, which was refreshed every second day. Alternatively, culture medium was sometimes supplemented with 0.5% DMF, 1% Nutridoma SP, and 0.5% FCS to induce granulocytic differentiation, as described by Pedruzzi *et al*. [17]. Cytospins from control and DMSO- or DMF-differentiated cells were submitted to Wright staining for counting and morphological characterization.

### Cytofluorimetric analyses

Cells were washed twice in PBS and 5 × 10^5 ^cells were incubated on ice for 30 min with anti-CD11b or isotype-matched control antibodies (0.25 μg/ml). After washing 3 times with PBS, the FITC-conjugated 2nd antibodies (0.5 μg/ml) were added and left to incubate for 30 min in the darkness before washing. Stained cells were analyzed (minimum of 10,000 cells) on a FACScan instrument (Becton Dickinson) using the CELLQuest software. For GFP expression, differentiated PLB-985 cells were washed twice in PBS before being analyzed by FACScan.

### Transient transfections

On the 5th day of granulocytic differentiation, PLB-985 cells were washed twice in pre-warmed PBS, and processed for transfection. When using the nucleofection technique, the cells were resuspended (5 × 10^6 ^cells/100 μl) in prewarmed Human Dendritic Cell Nucleofector Solution (Amaxa Biosystems, Köln, Germany) containing 5 μg of the plasmid of interest. Cells were incubated for 5 min at room temperature, transferred into a 2 mm-gap electroporation cuvette, and transfected in a Nucleofector instrument (Amaxa Biosystems) using the preconfigured Q-01 or U-15 settings. Nucleofected cells were washed once with pre-warmed RPMI 1640 medium containing 10% FBS, and then cultured in complete RPMI medium. When using the electroporation technique, day 5 granulocytic PLB-985 cells (5 × 10^6 ^cells/condition) were washed twice with pre-warmed PBS and resuspended in 400 μl of electroporation buffer (20 mM HEPES, 137 mM NaCl, 0.7 mM Na_2_HPO_4_) containg 5 μg of plasmid. The cells were transferred into a 4 mm-gap electroporation cuvette, and electroporated (270 V, 960 μF) using a Bio-Rad Gene Pulser instrument. Electroporated cells were washed with pre-warmed RMPI medium containing 10% of FBS, and cultured in a complete RPMI medium. These electroporation conditions represent optimized settings for maximal transfection efficiency and survival rate.

### Assays of β-galactosidase activity

Nucleofected cells were cultured for 6 h, washed twice with PBS, and resuspended in a buffer containing 40 mM TrisBase (pH 7.5), 150 mM EDTA, and 150 mM NaCl containing protease inhibitors. Cells were disrupted by three freeze/thaw cycles followed by one cycle of sonication (3 s, maximal power, on ice). Samples were cleared by centrifugation (10 min, 15,000 × g), and the supernatants were incubated at 37°C using 1-*O*-[2-nitrophenyl]-β-*D*-galactopyranoside as a substrate in a sodium phosphate buffer (pH 7.0), according to the supplier's instructions (Promega Technical bulletin #094).

### Luciferase assays

Nucleofected cells were cultured for 6 h in the presence or absence of stimuli, washed twice with PBS, and disrupted in Reporter Lysis Buffer following the manufacturer's instructions (Promega Corp., Madison, WI, USA). The lysates were cleared by centrifugation (12 000 g, 10 min), and the resulting supernatants were diluted using Luciferase Assay Reagent (Promega). Luciferase activity was then measured in a Sirius luminometer (Berthold Detection Systems, Pforzheim, Germany).

### Denaturing electrophoreses and immunoblots

Differentiated PLB-985 cells were resuspended in ice-cold PBS supplemented with protease inhibitors (10 μg/ml aprotinin, leupeptin, and pepstatin; 1 mM PMSF; 0.5 mM DFP) and phosphatase inhibitors (10 mM NaF, 1 mM Na_3_VO_4_, 10 mM Na_4_P_2_O_7_). A small aliquot was taken prior to centrifugation (300 g, 5 min, 4°C) for subsequent protein content determination, and an equal volume boiling sample buffer (2×) was added. Samples were briefly vortexed and immediately placed in boiling water for a further 3 min. Samples thus prepared were sonicated to disrupt chromatin, and stored at -20°C prior to analysis. All samples were electrophoresed on denaturing gels prepared according to the method of Laemmli [53]; equal loading was ascertained by adjusting sample volumes based on their respective protein content. Following SDS-PAGE, proteins were transferred onto nitrocellulose membranes, which were stained with Ponceau Red, destained, and then processed for immunoblot analysis, as previously described [31].

### Electrophoretic mobility shift assays (EMSA)

Cells were resuspended in ice-cold relaxation buffer (10 mM PIPES pH 7.30, 10 mM NaCl, 3.5 mM MgCl_2_, 0.5 mM EGTA, 0.5 mM EDTA, 1 mM DTT) supplemented the aforementioned protease and phosphatase inhibitors. Nuclear extracts were then prepared using a nitrogen bomb procedure, which we described previously [31,34]. The nuclear extracts were subsequently analyzed in EMSA for NF-κB, GRR, hSIE, and AP-1 binding as described earlier [31,34,36]. Except for the inclusion of 1 mM MgCl_2 _in the binding buffer, the binding conditions used for the C/EBP probe, 5'-tgcagaTTGCGCAATctgca-3', were identical to those used for NF-κB binding.

### Isolation of RNA and Ribonuclease protection assays

Neutrophils were incubated in the presence or absence of stimuli or inhibitors for the desired times, as indicated. Total RNA was extracted following a slightly modified Chomczynski & Sacchi procedure [54], and analyzed by ribonuclease protection assay as previously described [36], using multiprobe templates hCK3 or hCK5 from BD-Pharmingen (Mississauga, Ont, Canada).

### ELISA analysis of secreted proteins

Cells were cultured in 12-well plates at 37°C under a 5% CO_2 _atmosphere, in the presence or absence of stimuli and/or inhibitors, for the indicated times. Culture supernatants were carefully collected, snap-frozen in liquid nitrogen, and stored at -70°C. Cytokine concentrations were determined in in-house sandwich ELISA assays, using commercially available capture and detection antibody pairs (R&D Systems, BD-PharMingen). Detection limits using these assays varied between 3 and 10 pg/ml.

### Measurement of NADPH oxidase activity

Superoxide production by granulocytic PLB-985 cells was measured in whole cells by monitoring the reduction of cytochrome c in a ThermoMax microtiter plate reader (Molecular Devices). Briefly, cells cultured in PBS (supplemented with 5% FCS and 100 μM cytochrome c) were plated in a 96-well tissue culture-treated plate (2 × 105/well) and incubated at 37°C for the indicated times in the presence or absence of stimuli, prior to reading absorbance at 550 nm, and calculating the extent of superoxide generation.

## Abbreviations

AP-1, activator protein-1; C/EBP, CCAAT enhancer binding protein; GM-CSF, granulocyte-macrophage colony-stimulating factor; LPS, lipopolysacharide; Mip-1, Macrophage inflammatory protein-1; NF-κB, nuclear factor-κB; ROI, reactive oxygen intermediates; STAT, signal transducers and activators of transcription; TNF, tumor necrosis factor

## Authors' contributions

TE performed most of the experiments, analayzed the data, and drafted the manuscript. PPMcD conceived and designed the research project, provided the necessary funding, and edited the final manuscript. All authors read and approved the final manuscript.
